# Gene polymorphisms are associated with clinical outcome in Chinese resected laryngeal carcinoma patients

**DOI:** 10.18632/oncotarget.12323

**Published:** 2016-09-28

**Authors:** Peng Chen, Zhengshuai Chen, Jinglie Li, Hua Yang, Yuanyuan Zhu, Ning Zhang, Mengdan Yan, Yuan Shao, Chao Chen, Tianbo Jin

**Affiliations:** ^1^ The National Engineering Research Centre for Miniaturized Detection Systems, College of Life Science, Northwest University, Xi'an, Shaanxi 710069, China; ^2^ Key Laboratory of Resource Biology and Biotechnology in Western China (Northwest University), Ministry of Education, Xi'an, Shaanxi 710069, China; ^3^ Institution of Basic Medical Science, Xi'an Medical University, Xi'an, Shaanxi 710021, China; ^4^ Department of Otolaryngology-Head and Neck Surgery, The First Affiliated Hospital of Xi'an, Jiaotong University, Xi'an, Shaanxi 710061, China

**Keywords:** single-nucleotide polymorphism, laryngeal carcinoma, overall survival, prognosis, prognostic marker

## Abstract

We examined the multigenetic index on the progression of laryngeal carcinoma in Chinese population. This study aims to assess the effects of single nucleotide polymorphisms (SNPs) on survival of Laryngeal Carcinoma (LC) patients. Eighteen SNPs were selected and genotyped using the Sequenom iPLEX genotyping system in a cohort of 170 resected Chinese LC patients. Multivariate Cox proportional hazards model and Kaplan-Meier curve were used for the prognosis analysis. Overall, the median survival time (MST) was 38.00 months. The one, three and five year Kaplan-Meier survival rate was 0.847 ± 0.028, 0.572 ± 0.038 and 0.471 ± 0.041 respectively. The risks of death with the Hazard Ratio (HR) [95% confidence intervals] (CI) of 2.40 (1.15–4.50), 2.17 (1.45–3.25), 2.39 (1.58–3.62), 3.29 (2.10–5.18), respectively. There was significant associations between the SNPs and OS when the entire study population was examined. The rs1321311 TG genotype (vs.GG), rs2494938 AA genotype (vs. GG) and rs9363918 TG genotype (vs. GG) were associated with a worse prognosis for OS (adjusted HR = 1.64; 95%confidence interval = 1.07–2.51; *P* = 0.022, adjusted HR = 2.85; *P* =0.12; adjusted HR = 1.78; *P* = 0.009; respectively).

The results suggest for the first time that these gene polymorphisms may serve as an independent prognostic marker for LC patients.

## INTRODUCTION

Laryngeal carcinoma (LC) is one of the most frequently diagnosed malignant tumors in the Department of Otolaryngology - Head and Neck Surgery at the First Affiliated Hospital of Xi'an Jiaotong University [1]. LC can spread by directly invading adjacent structures, by metastasizing to regional cervical lymph nodes, or through the blood stream to more distant locations; among these, distant metastases in the lungs are the most common [2, 3]. LC resulted in 88,000 deaths in 2013, up from 76,000 deaths in 1990. Local recurrence after treatment is common, occurring in 10 to 50% of patients, and five-year survival rates in the United States are 60%. Adverse clinical prognostic factors in LC include advanced disease stage and poor performance status. However, clinical factors often do not accurately predict LC outcomes, and although a number of molecular markers accurately predict overall survival, none are currently used in the clinical setting [4].

The importance of genetic alterations in cancer has long been recognized, and although LC prognosis is not dependent on a single gene, it is affected by genetic differences between populations. The novel gene polymorphisms identified here that are associated with clinical outcomes in LC patients may help to improve personalized treatment strategies. Our findings suggest that these genes may be useful when screening for LC in Han Chinese populations and might aid in the discovery of additional molecular mechanisms that contribute to LC. [5] and Zhang *et* al.[6] were Genome scanningand PCR-RFLP methods have revealed that the H-RAS T81C mutation is associated with increased susceptibility to sporadic colorectal cancer. Mutations in P53, a human tumor suppressor gene, are involved in at least 1/2 of all tumors [7]. Single-nucleotide mutations are the most common genetic variations in the human genome, and the frequency of individual SNPs varies among different populations. Studying SNPs may therefore help explain individual and intra-population differences in susceptibility to complex diseases, drug resistance, and response to environmental factors. Recently, several separate genome-wide association scans have demonstrated that common SNPs, including those in the leucine rich repeat and fibronectin type III domain containing 2 (*LRFN2*), cyclin-dependent kinase inhibitor 1A (*CDKN1A*), brain-specific angiogenesis inhibitor 3 (*BAI3*), and parkin RBR E3 ubiquitin protein ligase (*PARK2*) genes, are associated with LC prognosis. All of these genes are located on Chromosome 5, 6, or 7 [8–10].

SNPs, which can serve as surrogates for patient's general genetic backgrounds, can predict therapeutic responses and prognoses [11, 12]. However, whether these variations influence clinical outcomes in LC patients remains unclear, and little is known regarding these associations specifically in Chinese patients. In this study, we assessed the association of 11 functional SNPs with survival in a cohort of 170 Chinese resected LC patients. To the best of our knowledge, this is the first study to investigate the predictive role of these SNPs for LC prognosis.

## RESULTS

### Patient characteristics and prognosis analysis

Between January 2002 and April 2013, a total of 170 laryngeal carcinoma patients (104 who underwent partial laryngectomy and 66 who underwent total laryngectomy) were included in this study. Patient characteristics are listed in Table 1 and Table S1. The median age was 60.75 years (range, 32 to 82 years). Of the 170 patients, 21.8% had stage I, 21.2% had stage II, 35.9% had stage III, and 21.2% had stage IV disease at the time of diagnosis. The median follow-up time was 38.00 months (range, 3 to 122 months). Of the 170 patients, 100 died before the final follow-up. Overall, the median survival time (MST) was 38.00 months, and the one, three, and five year Kaplan-Meier survival rates were 0.847 ± 0.028, 0.572 ±0.038, and 0.471 ± 0.041, respectively.

**Table 1:** Patient characteristics and prognosis

Variable			
			SD
Age (mean)		60.75	10.08
Median follow-up time (months)		38.00	30.65
Numbers of events	survival	70	
	death	100	
		Estimate	SE
Cumulative Proportion of patients surviving after the indicated time	1 year	0.85	0.03
	3 years	0.57	0.04
	5 years	0.47	0.04
Median survival time		48.00	9.56

As shown in Table 2, no significant associations were found between LC prognosis and age or lymph node detection. Differentiation, TN, clinical stage, and surgical method were associated with survival time, with hazard ratios for risk of death (95% CI) of 2.40 (1.15–4.50), 2.17 (1.45–3.25), 2.39 (1.58–3.62), 3.29 (2.10–5.18), respectively.

**Table 2:** Patient, treatment, and follow-up information

Patient characteristic		No. (%)	No. of deaths	Survival times	Cumulative proportion of patients surviving after the indicated time			*P*	HR (95.0% CI)
				Median ± SE	Estimate ± SE				
					1 year	3 years	5 years		
Age	< 60	80 (47.1)	47	73.00 ± 11.30	0.88 ± 0.04	0.597 ± 0.055	0.49 ± 0.06		
	≥ 60	90 (52.9)	53	30.00 ± 4.43	0.82 ± 0.04	0.574 ± 0.053	0.45 ± 0.06	1.161	0.78(1.72~0.46)
Differentiation	well differentiated	31 (18.2)	18	71.00 ± 28.29	0.87 ± 0.06	0.541 ± 0.091	0.50 ± 0.09	0.011*	
	moderately differentiated	125 (73.5)	70	59.00 ± 10.13	0.87 ± 0.03	0.620 ± 0.044	0.49 ± 0.05	0.794	0.93(0.56 ~1.57)
	poorly differentiated	14 (8.2)	12	15.00 ± 9.35	0.57 ± 0.13	0.214 ± 0.110	0.21 ± 0.11	0.020*	2.40 (1.15~ 5.00)
Clinical stages	T1-T2	102 (60.0)	52	77.00 ± 10.62	0.91 ± 0.03	0.663 ± 0.047	0.59 ± 0.05		
	T3-T4	68 (40.0)	48	32.00 ± 4.97	0.75 ± 0.05	0.437 ± 0.061	0.30 ± 0.06	0.000*	2.17(1.45 ~3.25)
	N0	116 (68.3)	61	71.00 ± 11.59	0.90 ± 0.03	0.670 ± 0.044	0.67 ± 0.04		
	N1-N2	54 (31.8)	39	26.00 ± 6.08	0.74 ± 0.06	0.361 ± 0.067	0.30 ± 0.07	0.000*	2.39(1.58~3.62)
	I-II	73 (42.9)	30	98.00 ± 15.57	0.97 ± 0.02	0.763 ± 0.050	0.69 ± 0.06		
	III-IV	97 (57.1)	70	32.00 ± 3.98	0.753 ± 0.04	0.429 ± 0.051	0.30 ± 0.05	0.000*	3.30 (2.10~5.18)
Treatment	Partial laryngectomy	104 (61.2)	50	73.00 ± 11.30	0.904 ± 0.03	0.678 ± 0.046	0.58 ± 0.05		
	Total laryngectomy	66 (38.8)	50	30.00 ± 4.43	0.76 ± 0.05	0.405 ± 0.061	0.30 ± 0.06	0.000*	2.35 (1.58 ~3.49)
	cervical lymph node dissection	37 (21.8)	19		0.270 ± 0.06	0.47 ± 0.09	0.47 ± 0.09		
	no cervical lymph node dissection	133 (78.2)	81	56.00 ± 10.04	0.89 ± 0.03	0.60 ± 0.04	0.48 ± 0.05	0.19	0.71(0.43 ~1.19)

Note:

* *P* < 0.05.

### Eighteen SNPs were associated with clinical outcome in LC patients

Genotype frequencies for the eighteen potentially relevant SNPs identified are shown in Table 3. We further explored the roles of 18 of these genetic polymorphisms in predicting LC prognosis. Four SNPs (rs1321311, rs2494938, rs9363918, and rs3016539) were associated with OS in the study population as a whole after adjusting for potentially confounding variables (age, differentiation, TN, clinical stages, surgical method, and lymph node detection) and with genotype as an indicator variable.

**Table 3:** Candidate SNPs

SNP	Chromosome	Position	Band	Alleles A/B	Gene(s)	Role
rs401681	5	1322087	5p15.33	T/C	*CLPTM1L*	Intron
rs6879627	5	2109901	5p15.33	T/C		
rs13361707	5	40791884	5p13.1	C/T	*PRKAA1*	Intron
rs367615	5	108948937	5q21.3	T/C		
rs647161	5	134499092	5q31.1	A/C		
rs9502893	6	1340189	6p25.3	C/T		
rs1321311	6	36622900	6p21.2	T/G	*SRSF3—CDKN1A*	
rs2494938	6	40536128	6p21.1	A/G	*LRFN2*	Intron
rs10484761	6	40802261	6p21.1	G/A		
rs9363918	6	69142008	6q12	T/G		
rs2057314	6	117819357	6q22.1	C/T	*DCBLD1*	Intron
rs4269383	6	156197502	6q25.3	A/G		
rs9365723	6	158435572	6q25.3	G/A	*SYNJ2*	Intron
rs7758229	6	160840252	6q25.3	T/G	*SLC22A3*	Intron
rs3016539	6	162236075	6q26	G/A	*PARK2*	Intron
rs2285947	7	21584088	7p15.3	A/G	*DNAH11*	Intron
rs39453	7	25133849	7p15.3	C/T		
rs10953615	7	109152711	7q31.1	G/A		

Abbreviations: SNP, Single-nucleotide polymorphism.

**Figure 1:**
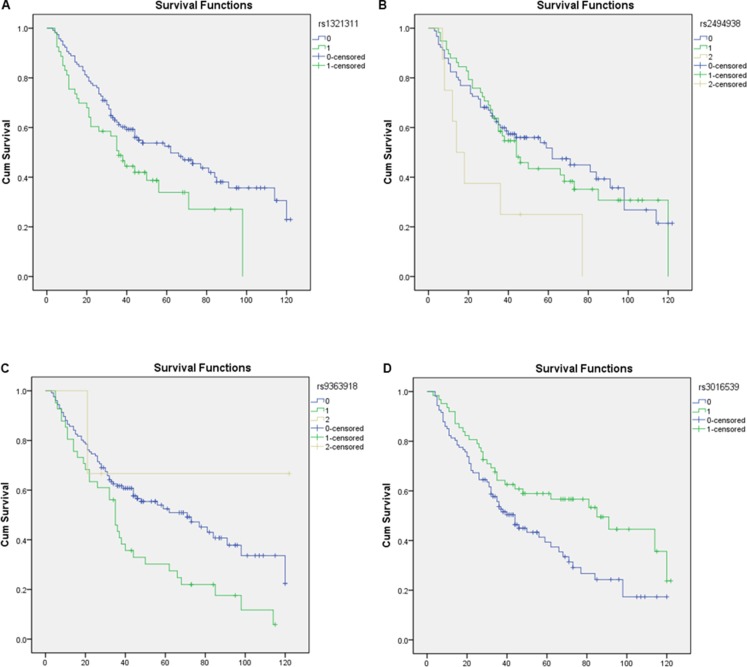
Kaplan-meier plots

As shown in Table 4 and Figure 1, HRs were higher for individuals with rs1321311, rs2494938, or rs9363918 polymorphisms. The rs1321311 TG (53_total_, 35_events_) genotype (vs. GG) (117_total_, 65_events_), rs2494938 AA (8_total_, 7_events_) genotype (vs. GG) (91_total_, 50_events_), and rs9363918 TG (41_total_, 34_events_) genotype (vs. GG) (126_total_, 65_events_) were associated with shorter OS (adjusted HR = 1.64, 95% confidence interval = 1.07–2.51, *p* = 0.022; adjusted HR = 2.85, 95% CI = 1.26 - 6.46, *p* = 0.12; adjusted HR= 1.78, 95% CI = 1.15–2.74, *p* = 0.009, respectively). The five year Kaplan-Meier survival rates were 0.524 ± 0.48 and 0.339 ± 0.77 for patients with rs1321311 G/G and G/A genotypes, 0.52 ± 0.57, 0.43 ± 0.69, and 0.25 ± 0.15 for patients with rs2494938 G/G, A/G, and A/A genotypes, and 0.53 ± 0.48, 0.30 ± 0.73, and 0.67 ± 0.27 for patients with rs9363918 G/G, T/G, and T/T genotypes, respectively. Additionally, HRs were lower for individuals with rs3016539 polymorphisms. The HR for the rs3016539 AG (62_total_, 31_events_) genotype (vs. AA) (107_total_, 68_events_) was 0.603, 95% Cl = 0.385–0.944, *p* = 0.027. The five year Kaplan-Meier survival rates for patients with the rs3016539 A/A and A/G genotypes were 0.394 ± 0.54 and 0.589 ± 0.63, respectively.

**Table 4:** Genetic polymorphisms and prognosis analysis

SNP_ID	Alleles A^a^/B	*N*	*N* of events	Median survival times (± SE)	Cumulative proportion of patients surviving after the indicated time (Estimate ± SE)			*P*^a^	Crude HR (95.0% CI)	*P*^b^	Adjusted HR (95.0% CI)
					1 year	3 years	5years				
rs1321311	G/G	117	65		0.89 ± 0.03	0.61 ± 0.05	0.52 ± 0.05				
	T/G	53	35	62.00 ± 13.26	0.76 ± 0.06	0.49 ± 0.07	0.34 ± 0.08	0.027	1.60 (1.05~2.43)	0.022*	1.64 (1.07~2.51)
	T/T	0									
rs2494938	G/G	91	50	62.00 ± 14.65	0.82 ± 0.04	0.60 ± 0.05	0.52 ± 0.06	0.066		0.038	
	A/G	58	36	44.00 ± 6.73	0.52 ± 0.06	0.59± 0.07	0.43 ± 0.07	0.663	1.10 (0.72~1.69)	0.952	1.01 (0.65~1.57)
	A/A	8	7	14.00 ± 4.24	0.63 ± 0.17	0.25 ± 0.15	0.25 ± 0.15	0.020	2.58 (1.16~5.72)	0.012*	2.85 (1.26~6.46)
rs9363918	G/G	126	65	71.00 ± 13.39	0.86 ± 0.03	0.62 ± 0.04	0.53 ± 0.05	0.014		0.025	
	T/G	41	34	35.00 ± 2.46	0.81 ± 0.06	0.43 ± 0.08	0.30 ± 0.07	0.006	1.80 (1.19~2.73)	0.009*	1.78 (1.15~2.74)
	T/T	3	1			0.67 ± 0.27	0.67 ± 0.27	0.474	0.48 (0.07~3.55)	0.515	0.51 (0.07~3.83)
rs3016539	A/A	107	68	44.00	0.81 ± 0.04	0.54 ± 0.05	0.39 ± 0.05				
	A/G	62	31	85.00	0.92 ± 0.04	0.64 ± 0.06	0.59 ± 0.06	0.015	0.59 (0.38~0.90)	0.027*	0.60(0.39~0.94)
	G/G	0									

Abbreviations: SNP, Single-nucleotide polymorphism; CI, confidence interval; HR, hazard ratio; Adjusted HR: adjusted by age, differentiation, clinical stage, and treatment.

* *p* < 0.05.

## DISCUSSION

Here, we examined whether 18 SNPs located on chromosomes 5, 6, and 7 were associated with survival in a cohort of 170 resected laryngeal carcinoma (LC) patients. The T/G genotype at SNP rs1321311, the A/A genotype at SNP rs2494938, and the T/G genotype at SNP rs9363918 were associated with shorter OS in LC patients. In contrast, the A/G genotype at SNP rs3016539 was associated with longer OS in LC patients. Although this was a preliminary, exploratory study, our results demonstrate for the first time that these gene polymorphisms may serve as independent prognostic markers in post-surgery LC patients. Once our results are confirmed, these SNPs could potentially be used in combination with traditional clinical prognosis factors to improve decisions regarding LSCC treatment.

The most common of the four OS-related SNPs, rs2494938, is located at 6p21.1 in an intron region of *LRFN2*, a member of the synaptic adhesion-like molecules (SALMs) family that interacts with the N-methyl D-aspartate (NMDA) receptor NR1 subunit through its extracellular or TM1 domains. This may at least partially explain the association between rs2494938 SNP and shorter survival in LC patients. Recent studies indicate that L*RFN2* is able to subvert hematopoietic differentiation to increase erythropoiesis, and may also be involved in the regulation of colony forming units and fibroblasts. Additionally, the minor alleles of rs2494938 are associated with an increased risk of lung cancer, non-cardia gastric cancer, and esophageal squamous-cell carcinoma in Han Chinese patients [13]. Wang *et al*. also found that rs2494938 SNP was associated with an increased risk of head and neck cancer in a Chinese Han population [14]. We found that two additional SNPs, at rs1321311 and rs9363918, were also associated with shorter survival in LC patients.

We also found that the rs1321311 TT genotype was associated with shorter survival in LC patients. The rs1321311 SNP, located at 6p21, is in linkage disequilibrium with a region that encompasses the *CDKN1A* gene, which encodes a potent cyclin-dependent kinase inhibitor that binds to and inhibits the activity of cyclin-CDK2 or -CDK4 complexes. CDKN1A regulates cell cycle progression at the G1 stage, and its expression is tightly controlled by the tumor suppressor protein p53; CDKN1A thus mediates p53-dependent cell cycle G1 phase arrest in response to a variety of stress stimuli [10]. Several reports indicate that CDKN1A, which is also associated with increased risk of esophageal cancer [15] and colorectal cancer [16], may be a useful predictor and target for cancer treatments involving cell cycle alteration.

The rs9363918 SNP is located upstream of the *BAI3* gene at chromosome 6q12. BAI3 may be involved in the inhibition of angiogenesis and glioblastoma development, and is associated with the development of brain and esophageal carcinoma and lung tumors [17–19]. The rs3016539 SNP is located in the *PARK2* gene, which is one of the largest human genes and codes for the protein parkin. Parkin tags damaged or unneeded proteins with ubiquitin molecules, marking them for degradation [20]. More than 200 *PARK2* gene mutations have been identified, and they contribute to Parkinson's disease and several forms of human cancer, including glioblastoma, colorectal cancer, lung cancer, ovarian cancer, breast cancer, renal cancer, and sporadic colorectal cancer. Because parkin is thought to act as a tumor suppressor, reduced parkin function might contribute to uncontrolled cell growth and division, promoting tumor formation. Bartek and Hodny reported that *PARK2* expression regulates cyclin-dependent kinases to ensure coordinated cell cycle progression and guard against tumorigenesis [21]. PARK2 is an important tumor suppressor in many different cancers [22, 23], and *PARK2* gene alterations are common in many human malignancies [18, 24]. *PARK2* is also associated with aggressive disease and poor clinical outcomes [25]; *PARK2* loss might trigger replication stress and fuel tumor progression by increasing genomic instability [25]. In our study, the rs3016539 A/G genotype was associated with longer survival in LC patients; it is possible that this mutation enhances the function of the *PARK2* gene.

The patients in this study were enrolled in Xi'an and adjacent areas. Due to the similarities in patient characteristics, this patient cohort may be especially useful for revealing population-specific influences of different SNPs; additionally, most patients continued to participate in the study throughout the follow-up period. Furthermore, because these patients received similar treatments, the confounding effects of different therapies that are common in cancer clinical outcome studies were minimized here. However, the limitations of this study should be considered when interpreting the results. Although there was sufficient statistical power to identify SNP-related differences in survival, the sample size was relatively small; larger studies should be conducted to confirm these results. In addition, future studies should focus on the mechanisms by which the SNPs identified here influence prognosis in Chinese LC patients.

The novel gene polymorphisms identified here that are associated with clinical outcomes in LC patients may help to improve personalized treatment strategies. Our findings suggest that these genes may be useful when screening for LC in Han Chinese populations and might aid in the discovery of additional molecular mechanisms that contribute to LC. Overall, our results strongly suggest that gene polymorphisms may be an independent prognostic marker in resected LC patients.

## MATERIALS AND METHODS

### Patient characteristics and treatment

170 laryngeal carcinoma (stages I to IV) patients who underwent laryngectomy at the First Affiliated Hospital of Xi'an Jiaotong University between January 2002 and April 2013 were included in this study. X-rays, CT, laryngoscopy, examination of laryngeal lesions, local cell smears, and pathology examinations were used for diagnosis. Eligible patients had pathologically-confirmed laryngeal carcinoma without distant metastases (M0). Each tumor was staged according to the 2010 UICC standard staging classification. All patients received surgery within 2 months of diagnosis, and no patient received anticancer treatment before surgery. All patients were Han Chinese males ranging in age from 32 to 82 years. All participants provided informed consent. Patients with recurring disease, or who had secondary laryngeal carcinoma with the primary cancer located elsewhere, were excluded. Patient information (age, sex, race, family history of cancer, and exposure) was recorded using questionnaires, and blood samples were collected for genotyping at the time of study entry.

Patient enrollment ended in 2013 to ensure adequate follow-up times for all participants; follow-ups continued for at least 2 years in all surviving patients. Complete outpatient records were obtained for the 170 patients, who were all treated at the First Affiliated Hospital of Xi'an Jiaotong University; complete genotype data was also available for all 170 patients.

### DNA extraction and genotyping

For each participant, 5 mL of venous blood was collected in sterile tubes with ethylenediaminetetraacetic acid at the time of enrollment. After centrifugation, samples were stored at −80°C until analysis. Genomic DNA was extracted from blood samples using a GoldMag-Mini Whole Blood Genomic DNA Purification Kit (GoldMag Co. Ltd. Xi'an City, China), and concentration was determined using a Nano Drop 2000 spectrophotometer (Thermo Fisher Scientific, Waltham, MA, USA).

The 18 SNPs analyzed here were identified in LC genome-wide association studies [8–10]. All of the SNPs had minor allele frequencies of > 5% in the HapMap for the Chinese Han Beijing population. Of these 18 SNPs variants, have been examined in Chinese populations. However, the results of previous genome-wide association studies and studies in Chinese populations are inconsistent. The Multiplexed SNP MassEXTENDED assay was designed using Sequenom MassARRAY Assay Design 3.0 Software (Sequenom Inc, San Diego, CA, USA) [26].

Genotyping of the 18 SNPs was performed using the Sequenom MassARRAY RS1000 system according to the manufacturer's instructions. Internal quality controls and negative controls were used to ensure genotyping accuracy, and 5% of the samples were randomly selected for repeat runs. Call rates for genotyping ranged from 99.0% and 99.4%. Two authors independently reviewed all agarose gels, data entry, and statistical analyses.

### Survival measurements

Time to event was defined as the duration of the period from the date of surgery to either event occurrence or the last date of the study (April 7, 2013), whichever came first. All causes of death were included when calculating overall survival (OS). Dates of death were obtained and cross-checked using at least one of the following three methods: Social Security Death Index, the First Affiliated Hospital of Xi'an Jiaotong University tumor registry, and in- or outpatient medical records confirmed by the patient's primary care physician and/or family. Surviving patients were censored using the date of last contact. This date was verified by inpatient and outpatient medical records, and/or confirmation with the patient's primary care physician and/or family.

### Statistical analysis

Overall survival was defined as the time from surgery to death due to laryngeal carcinoma. All genotypes for each SNP were included in the analysis. Hazard ratios (HRs) and 95% confidence intervals (CIs) were estimated separately for each SNP using the Cox proportional hazard model and after adjusting for age, tumor differentiation, TNM stage, surgical method, and lymph node detection. Cumulative proportions of patients surviving were estimated using the Kaplan–Meier statistical method. The log-rank test was used to evaluate the possible impact of genetic polymorphisms, separately and in combination, on OS. Additional clinical factors were also included in the analysis. *P* < 0.05 was considered statistically significant, and all analyses were conducted using the SPSS 17.0 statistical package (SPSS, Chicago, IL).

## SUPPLEMENTARY MATERIALS TABLES


