# Supplementary material for: Gene polymorphisms are associated with clinical outcome in Chinese resected laryngeal carcinoma patients
Source: Oncotarget. 2016 Sep 28;7(44):71703–9. doi: 10.18632/oncotarget.12323 (PMC5342113; doi:10.18632/oncotarget.12323)
Supplement: Supplementary file 1 [file oncotarget-07-71703-s001.pdf]

Gene polymorphisms are associated with clinical outcome in Chinese resected laryngeal carcinoma patients

Supplementary Materials

Supplementary Table S1: Patient, treatment, and follow-up characteristics

Patient Characteristic		No. of Patients	%
Age	< 60	80	47.1
	≥ 60	90	52.9
differentiated	welldifferentiated	31	18
	moderatelydifferentiated	125	70
	poorly differentiated	14	12
clinical stages	T1	40	23.5
	T2	62	36.5
	T3	50	29.4
	T4	18	10.6
	N0	116	68.2
	N1	30	17.6
	N2	24	14.1
		37	21.8
		36	21.2
		61	35.9
		36	21.2
modus operandi	vertical hemilaryngectomy	54	31.8
	Coronary laryngectomy	2	1.2
	Partial excision of the larynx	1	.6
	total laryngectomy	66	38.8
	horizontal hemilaryngectomy	43	25.3
	horizontal vertical hemilaryngectomy	4	2.4
	cervical lymph node dissection	37	21.8
	no cervical lymph node dissection	133	78.2
outcome	survival	70	41.2
	death	100	58.8
